# Kinetics of Quality Degradation and Water Removal During Air Drying of Osmodehydrated Oyster Mushrooms Impregnated with *Rosa damascena* Distillation By-Products

**DOI:** 10.3390/foods14091543

**Published:** 2025-04-28

**Authors:** Natalia A. Stavropoulou, Andriana E. Lazou, Maria C. Giannakourou

**Affiliations:** 1 Laboratory of Chemistry, Analysis & Design of Food Processes, Department of Food Science and Technology, School of Food Sciences, University of West Attica, Agiou Spyridonos St., Egaleo, 122 43 Athens, Greece; nstavropoulou@uniwa.gr (N.A.S.); alazou@uniwa.gr (A.E.L.); 2 Laboratory of Food Chemistry and Technology, School of Chemical Engineering, National Technical University of Athens, Zografou Campus, Iroon Polytechniou, 157 80 Athens, Greece

**Keywords:** browning, drying kinetics, mathematical modeling, mushrooms, osmosis, *Rosa damascena* waste water

## Abstract

Mushrooms are a valuable food in the human diet due to their superior nutritional properties. However, mushrooms’ short shelf life presents a challenge for their commercial application. Mushrooms’ air drying kinetics were determined, and the impact of prior osmotic dehydration was quantitatively evaluated. Additionally, the sustainable utilization of *Rosa damascena* distillation wastewater, rich in phenolics, was explored. Samples were impregnated with hypertonic solutions including rose wastewater, glycerol, salt and calcium chloride, and air-dehydrated at 40, 55, and 70 °C. Texture and color changes were determined during drying. Seven acknowledged mathematical models were successfully applied to describe the drying kinetics, with the effect of process temperature being incorporated into the drying constant. The simplest first-order model is deemed adequate for describing moisture reduction and quality degradation. Pretreatment significantly reduced the drying time to reach a final moisture content of 10% w.b, especially at 70 °C, where the reduction obtained was more than 40% (5 h for untreated vs. 2.5 h for pretreated samples). At the end of drying, pretreated samples reached lower values of water activity and maintained their color better (25–50% improvement). This study aims to provide a basis for producing a novel, mushroom-based, nutritionally fortified dry snack, following results confirmed by a sensory examination.

## 1. Introduction

Oyster mushrooms are the second most cultivated mushrooms in the world, valued for their nutritional, medicinal and income contributions. They are high in protein (up to 30–40% protein, dry weight), dietary fibers, and ergosterol and low in fat and sodium, making them a healthy food option. A major challenge is that oyster mushrooms have the shortest shelf life of any cultivated mushroom due to their high water content, fragile nature (due to the lack of a protective layer on the skin), and susceptibility to bacterial and fungal contamination [1]. Generally, mushrooms are highly perishable products that can be stored only for a few days (or 24 h at room temperature). To extend their shelf life, fresh mushrooms must be processed [2,3]. Drying is the most commonly used preservation method to extend the shelf life of foods because it is a cost-effective method to reduce the moisture content. Drying can prevent the growth of microorganisms, delay enzyme activity, and slow down degradative reactions that require water as a medium [4,5]. It also reduces the weight for transportation and requires less storage space [6].

However, hot air drying process has several important drawbacks. These include low energy efficiency, long drying times, and quality deterioration, as well as an undesirable modification of the flavor-containing components so that dried mushrooms show different sensory characteristics from those of fresh ones [2,7]. As highlighted by numerous researchers, exposure to higher temperatures for extended periods can result in a substantial deterioration of the flavor, color, and nutritional content of dried mushrooms due to the reduction in certain valuable components that are prone to oxidation at high temperatures. In addition, traditional thermal processing methods are energy intensive [3,5,8].

Dried mushrooms have recently been used as a key ingredient in a wide range of food formulations, including instant soup premixes, snack seasonings, pasta, pizza, salads, and meat and rice dishes [5,8]. To mitigate the significant quality degradation of dried mushrooms and enhance the efficiency of thermal processing, researchers have extensively examined innovative technologies like infrared [5,9], microwave [2,8], pulsed electric field, and ultrasound [10]. In Moutia et al. [11], a review analyses the impact of major dehydration techniques, including sun drying, hot air drying, microwave drying, and freeze drying, on the nutritional and microbial profiles of dried mushrooms. Infrared drying has been used as an alternative drying process for mushrooms. Wang et al. [9] report that infrared drying (IR) has been shown to exhibit a faster drying rate than air drying, since the absorption of infrared energy results in the direct heating of materials without warming the drying medium. On the other hand, microwave, pulsed electric field, and ultrasound processing techniques have been applied for mushroom drying as pretreatments. Das and Arora [8] posit that the use of microwave technology in the drying process is more efficacious than the conventional hot air drying method. The outcomes of this study demonstrate a reduction in drying time and an enhancement in product quality. The quality parameters that were enhanced include water activity reduction, lighter color, and a higher rehydration ratio. As pointed out by Mirzaei-Baktash et al. [10], the use of pulsed electric fields or ultrasound as a pretreatment resulted in a reduction in specific energy consumption and an abridgement of the drying time. Investigating the drying rate of PEF pretreated mushroom samples, they demonstrated an absence of the constant drying period, with the entire drying occurring in the falling rate period. In a recent paper by Das et al. [12], three different drying methods, namely hot air drying, dehumidified drying, and freeze drying, were used to dry Indian white button mushrooms (*Agaricus bisporus*) to produce a powder for ready-to-cook meals. The results revealed that the dried mushroom powder obtained from the dehumidified drying method showed a conservation of nutrients and color, and satisfactory physicochemical characteristics also.

On the other hand, to improve the quality attributes of the final dry foodstuff, several pretreatments can also be applied prior to the main drying step, and a comprehensive summary of commonly used techniques for mushroom drying is provided in Yang et al. [13]. In that review, thermal (steam, microwave and ohmic blanching) and nonthermal methods (ultrasonication, osmotic dehydration, cold plasma, pulsed electric fields) are presented, along with their advantages and limitations. Amongst them, the application of osmotic dehydration prior to air drying is proposed as an effective and energy/cost efficient alternative to reduce the drying time of mushrooms and maintain their quality attributes. Moreover, osmotic treatment can extend the retention of pigments and reduce the browning that occurs due to natural oxidative processes [14]. Tyrosinase, which belongs to the PPO (polyphenol oxidase enzyme) family, causes the enzymatic oxidation of phenolic compounds, leading to browning in mushrooms [15]. At this point, one should note that osmotic treatment alters the profile of the fresh tissue due to the solid gain from the osmotic solution, which does not always present positive effects on the nutritional and physical–mechanical properties of foods, presenting advantages or disadvantages to the final product [16]. In this context, the knowledge of the impact of controlling process parameters, i.e., process temperature and duration, osmotic solution composition and concentration, on the kinetics of water loss and solid uptake is important to design a product with specific attributes (tissue engineering) and to optimize the procedure [17]. The impact of OD as a pretreatment for drying has been explored in the recent literature for a variety of fruits and vegetables, with the case of mushrooms being underexplored. Drying involves exposure of the food to high temperatures for long periods, promoting undesirable quality changes and nutritional degradation. Therefore, the reduction in total drying time accomplished with a preliminary OD step could improve the quality. Furthermore, since OD is carried out at mild temperatures, the drying step can be applied at lower than the temperatures usually used, and the decrease in the drying time contributes to lower energy consumption and better quality retention [18,19,20]. A detailed review of the influence of osmotic dehydration on the drying process is presented in Abrahão and Corrêa [16], where the main step of the air drying process is implemented at mild temperatures (lower than 70 °C), as shown in Mandala et al. [21] (50 °C), Pavkov et al. [22] (40, 50, and 60 °C), Romdhane et al. [23] (40 and 60 °C), Sahin et al. [24] (55, 65 and 75 °C), Dermesonlouoglou et al. [25] (60 °C), Guiamba et al. [26] (50 °C and 70 °C), etc.

While many researchers have explored various methods of drying different types of mushrooms, aiming to prevent quality degradation, the combination of osmotic dehydration and air drying has not been extensively investigated in mushrooms. This method is currently more popular in other food categories, such as fruits, and only a limited number of studies have been conducted on mushrooms. Tolera et al. [27] studied the nutritional quality of oyster mushrooms, as affected by osmotic pretreatments and drying methods. The results showed that OD pretreatment can address the perishability issue of mushrooms. Torringa et al. [28] studied osmotic dehydration as a pretreatment, prior to the microwave–hot air drying of mushrooms. The researchers reported that osmotic pretreatment leads to a slightly shorter drying time, improves rehydration properties, and reduces shrinkage.

In order to ensure an effective and successful drying process, it is first necessary to evaluate the drying kinetics and assess the effect of certain processing parameters on the final product quality. Mathematical models have been used to build new drying systems, enhance existing ones, and even optimize the drying process itself. In order to predict product quality and optimize drying processing variables, food drying kinetics incorporates the use of fundamental mathematical models. In the literature, many researchers use several thin-layer drying models to illustrate the drying characteristics of different kinds of food [29,30]. The mathematical models used in the field of the food drying industry can be categorized into two groups, the semi theoretical models and the empirical models. Semi theoretical models are derived from theoretical principles, often based on Fick’s laws of diffusion or modified forms of simplified models. Some of the key semi-theoretical models include the Lewis model, Page model, Modified Page model (II) and (III), Henderson and Pabis Model, Logarithmic model, Wang and Singh, Weibull model, etc. Empirical models are based on experimental data and do not necessarily derive from theoretical principles. They are often simpler and require less time to develop. Some of the empirical models include the Diamante model, Wang and Singh model, Thompson model, Logistic model, etc., [21,31,32,33]. Models that have been successfully used in mushroom drying include the Page model, Modified Page model, Midilli model and Weibull model [31,34,35,36].

Over the last decade, there has been a constant increase in the production of essential oils from aromatic plants and flowers to meet the demands of various industries, such as the cosmetic and food industries. However, the processing of essential oils creates a significant amount of by-products, including liquid fractions known as hydrolates and a solid fraction that includes the biomass remaining after the distillation process. For both economic and environmental reasons, it is important to find ways to valorize or reuse these by-products. *Rosa damascena* Mill of the Rosaceae family is reported to be of the most popular ornamental flowers with recognized pharmacological properties, widely used in the food and perfume industry. These substrates are a rich source of bioactive compounds such as phenolic and polyphenolic compounds, carotenoids, tannins, catechins, and terpenoids, which exhibit antimicrobial and antioxidant activity [37,38,39]. Rose hydrosols have been shown to reduce the enzymatic browning of fresh-cut products due to their antioxidant properties inhibiting the activity of PPO [28]. These by-products have also been successfully applied to bacon, sea bass, and mushrooms, in order to improve their quality and nutritional value and extend their shelf life [37,38,40].

The objective of this study is to investigate the air drying kinetics of fresh and osmo-dehydrated oyster mushroom (*Pleurotus ostreatus*) slices processed with alternative osmotic agents (rich in bioactive compounds). The effect of drying temperature on kinetics was investigated, and several mathematical models were used to describe the mass transfer during the process. Moreover, textural and color characteristics, including hardness, total color difference, and browning index, were examined during drying and described through a pseudo zero- and first-order kinetic model, respectively. The purpose was to obtain a methodology for designing a novel, nutritionally enriched, dried mushroom-based snack, optimizing a two-step drying procedure.

## 2. Materials and Methods

### 2.1. Raw Material

Fresh, edible oyster mushrooms with an initial moisture content of 90.85 ± 1.34% (wet basis, w.b.) were obtained from the local company “DIRFIS MUSHROOMS”. The moisture content was calculated after vacuum drying at 70 °C (Heraeus Instruments Vacutherm, ThermoScientific, Waltham, MA, USA) for 24 h, according to the official AOAC Method 934.06 for measuring water content [41]. Each measurement was repeated three times to obtain the average value and standard deviation. Mushrooms of uniform size were selected, cleaned with tap water, and dried on blotting paper. Samples were then immersed in freshly prepared osmotic solutions.

### 2.2. Osmotic Dehydration Pretreatment

Oyster mushrooms were osmotically treated at optimum conditions, as calculated in previous work by our research team [40]. Briefly, mushroom samples were immersed in concentrated solutions of food-grade glycerol (Honeywell Specialty Chemicals Seelze GmbH, Seelze, Germany) at concentrations of 30, 40, and 50%, 5% NaCl and 1.5% calcium chloride (CaCl_2_) (Chem-Lab NV, Zedelgem, Belgium), at 30–40–50 °C and for up to 190 min of immersion time. In this work, the Response Surface Methodology (RSM), coupled with Box–Behnken design and desirability functions, were used to optimize the process (Minitab^®^ 17.1.0, LEAD Technologies, Inc., Charlotte, NC, USA).

**Figure 1:**
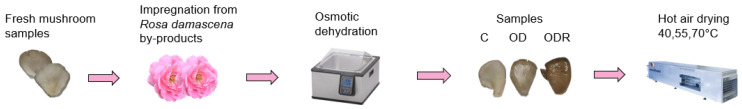
Experimental setup, showing the successive steps of mushroom processing.

The optimization criteria included the minimization of a_w_ and the maximum retention of the initial lightness (expressed as lightness preservation (*L*/*L*_0_)), with a minimum value of 0.8, based on a preliminary sensory test. The process of optimization and experimental design is described in detail by Stavropoulou and Giannakourou [40]. The optimum conditions were found to be 38.7 °C (temperature of osmosis), 30% glycerol (concentration of the osmotic solution), and 130 min (duration of osmosis). To minimize dilution of the medium by water removal, the sample-to-solution ratio was kept at a ratio of 1:15 (*w*/*w*). To enrich mushrooms with polyphenolic compounds, samples were immersed in the *Rosa damascena* distillation wastewater at a ratio of 1:10 for 10 min, before OD at the optimized conditions, as described by Stavropoulou and Giannakourou [40]. The initial content of total phenols (*TPC*) in *Rosa damascena* by-products was measured by the modified Folin–Ciocalteu micromethod, as reported by Andreou et al. [42]; it was found to be 1539.14 ± 187.43 mg GAE/L. The experimental process implemented is illustrated in the graph below (Figure 1).

### 2.3. Air Drying Process

The osmotically pretreated mushroom pieces were further dehydrated in an experimental air dryer (Armfield, Ltd., Ringwood, UK). The objective was to study the effect of osmotic dehydration pretreatment on the moisture loss rate and on the quality characteristics of the dried mushrooms. To achieve this, samples were placed in a perforated tray and the drying process was carried out at three temperature levels of 40, 55, and 70 °C until constant weight. The air velocity was kept constant at 0.9 m/s. Sample weight was recorded every 5 min for the first hour, every 10 min for the next 1 h, every 15 min for the next 3 h, and then every 30 min until a constant weight was achieved. Sample a_w_ value, color, and texture were measured every 30 min for the first 2 h and every 60 min for the next hours until the end of the drying process. Three replicate samples were removed and measured each time, and the average values and standard deviation were calculated.

### 2.4. Determination of Quality Characteristics During Air Drying Process

#### 2.4.1. Water Activity

The water activity of the mushroom samples was quantified by an a_w_-meter (AquaLab Dew Point Water Activity Meter 4TE, METER Group, Inc., Pullman, WA, USA) throughout the drying process.

#### 2.4.2. Color

During the air drying process, color was measured with a tristimulus chromatometer (Handy Color Tester, Model H-CT, SUGA Test Instruments). Color measurements of the mushroom samples were obtained at three distinct points on their surfaces. At each time point, three mushroom samples were measured. The CIELAB color scales were used, with coordinates L*, a*, b*. The total color change Δ*E* was calculated according to Equation (4) [43,44]:

(1) $$\Delta Ε=\sqrt{{\left(L_{t}^{*}-L_{0}^{*}\right)}^{2}+{\left(a_{t}^{*}-a_{0}^{*}\right)}^{2}+{\left(b_{t}^{*}-b_{0}^{*}\right)}^{2}}$$

Browning index (*BI*) was also calculated according to Equation (5) [45,46,47]:

(2) $$BI=\frac{\left[100\left(A-0.31\right)\right]}{0.17}$$

where

(3) $$A=\frac{a^{*}+1.75L^{*}}{5.645\;L^{*}+\;a^{*}-3.012b^{*}}$$

where L*, a* and b* represent the level of lightness, redness, and yellowness of the samples, respectively. Subscripts “*t*” and “0” refer to time t and zero time, respectively.

#### 2.4.3. Textural Properties

A texture analyzer (TA-XT2i of Stable Micro Systems, Godalming, UK) was used for texture analysis of the mushroom samples and a TPA (Texture Profile Analysis) test was performed. The experiment was carried out according to the method described in Stavropoulou et al. [48]. Briefly, the test was performed on a non-lubricated flat platform using a 6 mm cylindrical compression probe and a 25 kg load cell under the following instrument parameters: pre-test speed—5 mm/s; test speed—2 mm/s; and post-test speed—5 mm/s at 50% deformation. For this purpose, the exact thickness of each sample was measured, and the 50% deformation was calculated. Hardness (F_max_), defined as the maximum force of the first compression on the TPA curve [49,50], was calculated. All measurements were performed in triplicate.

### 2.5. Mathematical Modeling of Drying Kinetics and Properties

#### 2.5.1. Mass Transfer Kinetics

A first-order reaction kinetic model (Newton model) was used to describe drying kinetics and predict the drying rate, with the rate constant being a function of the process temperature, as follows [29,51]:

(4) $$-\frac{dX}{dt}\;={\;k}_{x}\cdot (X-X_{e})$$

where *X* is the material moisture content (dry basis) during drying (kg water/kg dry solids), *X_e_* is the equilibrium moisture content of dehydrated material (kg water/kg dry solids), *k_x_* is the drying rate (min^−1^), and t is the time of drying (min). The drying rate is determined by the slope of the falling rate of the drying curve.

The solution of Equation (1) derives the following expression:

(5) $$X\;=\;X_{e}+(X_{i}-X_{e})\cdot e^{-k_{x}\cdot t}$$

The effect of process temperature during air drying was included in the model parameters as follows:

(6) $$k_{X}=k_{0,x}\cdot {\left(\frac{T}{55}\right)}^{k_{1x}}$$

where *k*_0,*x*_ is a constant (min^−1^), *k*_1,*x*_ is a dimensionless constant (-), and *T* is the dry bulb temperature of air (°C). The temperature was divided by 55 since this is the central temperature value of air drying used in the experimental design [29].

Non-linear regression analysis was used for parameter estimation using SYSTAT 10.2 (Stat. Soft Inc., Tulsa, OK, USA).

An alternative way to express mass transfer during drying is through the dimensionless moisture ratio, frequently presented in studies regarding the drying rate. In this context, the moisture ratio (*MR*) of mushroom pieces during drying experiments was calculated using the following equation:

(7) $$MR=\frac{X-X_{e}}{X_{0}-X_{e}}$$

The values of ‘*X_e_*’ are much smaller than those of ‘*X*’ or ‘*X*_0_’. Consequently, the error involved in the simplification is deemed to be negligible [52]. Therefore, the moisture ratio was calculated as follows:

(8) $$MR=\frac{X}{X_{0}}$$

Six well-known thin-layer drying models (see Table 1) were used to create drying curves for model selection, based on the change in the dimensionless *MR* index vs. drying time. In these models, *k_MR_* is defined by a similar equation, Equation (6):

(9) $$k_{MR}={\;k}_{0,MR}\cdot {\left(\frac{T}{55}\right)}^{k_{1MR}}$$

where *k*_0,*MR*_ is a constant (min^−1^), *k*_1,*MR*_ is a dimensionless constant (-), and *T* is the dry bulb temperature of air (°C).

The selection of the most appropriate model was determined by evaluating four key parameters: the coefficient of determination (*R*^2^), the reduced sum of squares (*SSE*), the root mean square error (*RMSE*), and the reduced Chi square (χ^2^), as outlined in Equations (10)–(13), respectively [31,32,34].

(10) $$R^{2}=1-\frac{\sum_{i=1}^{N\;}{\left({MR}_{exp,ii}-{MR}_{pre,i}\right)}^{2}}{\sum_{i=1}^{N}{\left({MR}_{exp,i}-{MR}_{exp,average}\right)}^{2}}$$

(11) $$SSE=\sum_{i=1}^{N}{\left({MR}_{exp,i}-{MR}_{pre,i}\right)}^{2}$$

(12) $$RMSE={\left[\sum_{i=1}^{N}{\frac{1}{N}\left({MR}_{exp,i}-{MR}_{pre,i}\right)}^{2}\right]}^{\frac{1}{2}}$$

(13) $$\chi^{2}=\sum_{i=1}^{N}\frac{{\left({MR}_{exp,i}-{MR}_{pre,i}\right)}^{2}}{N-m}$$

In Equations (10)–(13), *MR*_*pre*,_*_i_* is the ith predicted moisture ratio, *MR*_*exp*,_*_i_* is the ith experimental moisture ratio, *N* is the number of observations, and *m* is the number of parameters in the model.

**Table 1:** Frequently used thin-layer drying curve models.

Model	Model Equation	References
**Lewis**	$MR=exp(-k_{MR}t)$ (14)	[51,53]
**Page**	$MR=exp(-k_{MR}t^{n})$ (15)	[33,54]
**Modified Page**	$MR=exp\left[-{\left(k_{MR}t\right)}^{n}\right]$ (16)	[55,56]
**Henderson and Pabis**	$MR=\alpha exp\left(-k_{MR}t\right)$ (17)	[34,57]
**Weibull**	$MR=exp\left[-{\left(\frac{t}{k_{MR}}\right)}^{n}\right]$ (18)	[58]
**Midilli**	$MR=\alpha exp\left[-k_{MR}t^{n}\right]+bt$ (19)	[35,59]

where *k_MR_* is the drying constant (min^−1^), calculated by Equation (9), and *n*, *a*, and *b* are empirical constants.

#### 2.5.2. Color and Texture Kinetic Modeling

Similarly to Equation (1), a first-order equation (fraction model) is also proposed to describe color changes, namely the evolution of the Browing Index (*BI*) and total color (Δ*E*) during drying, as follows:

(20) $$\frac{dBI}{dt}=k_{BI}\cdot \left({BI}_{e}-BI\right)⟹BI={BI}_{e}+(BI_{i}-{BI}_{e})\cdot e^{-k_{BI}\cdot t}$$

(21) $$\frac{d∆E}{dt}=k_{∆E}\cdot \left({∆E}_{e}-∆E\right)⟹∆E={∆E}_{e}-{∆E}_{e}\cdot e^{-k_{∆E}\cdot t}\;\;\;\;\;$$

where *BI_ι_* and *BI_e_*/Δ*E_e_* are the parameter value at time zero and at equilibrium, respectively, k_BI/_k_ΔE_ is the rate constant (min^−1^) of the particular color parameter, and t is the air drying time (min).

On the other hand, the most representative textural attribute, expressed by hardness (*F*), has been found to follow a pseudo-zero-order reaction:

(22) $$\frac{dF}{dt}=-k_{F}⟹F=F_{e}+k_{F}\cdot t$$

where *F* is the parameter of hardness (N), *F_e_* is the equilibrium value (N), *k_F_* is the rate constant (min^−1^) of the hardness parameter, *t* and is the time of the air drying (min).

Similarly to Equation (6), the effect of the process temperature during air drying was included in the model parameters as follows:

(23) $$k_{BI}=k_{0,BI}\cdot {\left(\frac{T}{55}\right)}^{k_{1BI}}$$

(24) $$k_{\Delta E}=k_{0,\Delta E}\cdot {\left(\frac{T}{55}\right)}^{k_{1\Delta E}}$$

(25) $$k_{F}={\;k}_{0,F}\cdot {\left(\frac{T}{55}\right)}^{k_{1F}}$$

where *k*_0,(*BI*, Δ*E*)_ are constants (min^−1^), *k*_1,(*BI*, Δ*E*)_ are dimensionless constants (-), and *T* is the dry bulb temperature of air (°C).

### 2.6. Data Analysis

Experiments were performed in multiple repetitions, as indicated per case. Analysis of variance (ANOVA) at a significance level of 95% was used to investigate if there were any significant differences amongst parameter values (STATISTICA^®^ 7.0, StatSoft Inc., Tulsa, OK, USA). Significant differences were calculated according to Duncan’s post hoc multiple range test (a = 0.05). Non-linear regression analyses and kinetic parameter estimation of all the models implemented, along with their ±95% confidence intervals, were performed using SYSTAT 10.2.

## 3. Results and Discussion

### 3.1. Effect of Drying Temperature on Drying Kinetics

#### 3.1.1. Water Content Reduction

**Figure 2:**
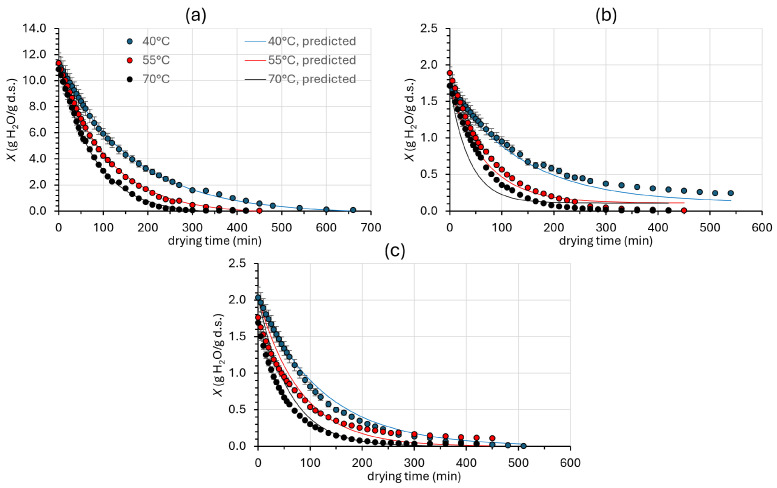
Drying kinetics of (**a**) fresh (Control), (**b**) osmotically dehydrated (OD), and (**c**) osmotically dehydrated and previously impregnated with bioactive compounds from *Rosa damascena* distillation wastes (ODR) mushroom samples at 40, 55, and 70 °C, where X is moisture content in g H_2_O/g dried solids (dry basis). Solid lines represent the model fitting (Equations (5) and (6)). Error bars depict the standard deviation out of three replicates.

Figure 2 illustrates the effect of air drying temperature (40, 55, and 70 °C) and drying duration on the moisture content of the mushroom pieces. The lines in Figure 2 depict the predicted moisture content values for each sample (based on Equations (5) and (6)), while the markers represent the corresponding average experimental values.

The drying rate at the beginning of the process is higher for all samples and then slows down until the moisture content reaches an equilibrium at all drying temperatures. The drying rate is slower at lower temperatures, while it increases as the temperature increases for both treated and untreated samples. This result is consistent with previous studies on dried Pleurotus mushrooms [60,61]. Nevertheless, the influence of air temperature was less important from 55 to 70 °C. Furthermore, judging from the shape of the curves at all the drying temperatures investigated, a constant drying rate period was not detected, and only one diffusional, falling rate period was observed until it reached its equilibrium moisture content [62], which is in agreement with the drying results for a variety of fruit tissues. For many plant products, the constant drying period is either insignificant or it does not exist because the internal heat and mass transfer rates are determining factors of the rate at which moisture can be available at the exposed evaporating surface.

As shown in Figure 2, there is a significant difference in the initial moisture content of the samples, depending on whether they have been OD pretreated or not. The initial moisture of the samples during air drying is actually equal to the final moisture content obtained after osmotic pretreatment at 38.7 °C, with 30% glycerol, 5% NaCl, and 1.5% calcium chloride (CaCl_2_) for 130 min (optimized conditions of the OD process). The initial moisture (d.b.) for osmodehydrated samples was 1.77 ± 0.10 gH_2_O/g d.s. for OD, 1.83 ± 0.18 gH_2_O/g d.s. for ODR, and 11.18 ± 0.27 gH_2_O/g d.s. for fresh mushrooms. As a result, the osmotically pretreated samples reach the equilibrium faster when compared to control samples, significantly reducing the time required for mushroom drying. Finally, as expected, no significant differences (*p* > 0.05) in moisture content were observed between the OD and ODR samples during drying.

When comparing control samples with OD/ODR, the time required to reach a final moisture content of 10–13% w.b. (corresponding to approximately 0.11–0.15 g H_2_O/g dry solids), which is characteristic of air-dried products and ensures their long-term preservation [8,63], was reduced by more than two times based on model predictions. More specifically, at an air drying temperature of 70 °C, the time required for drying was reduced from 6 h (control) to 2.7 h (OD) and 2.5 h (ODR), indicating a more pronounced time reduction, compared to lower temperatures. At drying temperatures of 40 and 55 °C, the untreated samples required more than 11 and 7.5 h, respectively, to reach a moisture content of less than 0.11–0.15 g H_2_O/g dry solids; on the contrary, the osmodehydrated samples required 7.3 and 4.2 h, respectively.

No similar research on the application of osmotic dehydration prior to air drying has been conducted on mushrooms; however, the results are in good agreement with the relative observations of various plant products. More specifically, Biswas et al. [30] studied the osmotic dehydration of pineapple combined with hot air drying. Fresh pineapple slices were immersed in four osmotic solutions (1% trehalose, 2% NaCl, 10% sucrose, and 10% fructose) for 30 min. After pretreatment, samples were dried at 50, 55, and 60 °C. The results showed that pretreated pineapple slices dried faster than the untreated ones, at a constant drying temperature. Malekie et al. [64] studied an osmotic impregnation technique combined with ultrasonic pretreatment to carrot slices followed by hot air drying. For the OD, they used sucrose at concentrations of 40, 50, 60% and process times of 30, 60, 90, and 120 min and at a constant temperature of 50 °C. The samples osmotically pretreated under the optimum conditions were dried at 60, 70, and 80 °C using a hot air drier. The results demonstrate that the drying temperature had a significant effect on the final moisture content. In more specific terms, the rate of moisture content removal increased as the drying temperature increased, which resulted in a reduction in the drying time. Kadir et al. [65] studied the osmotic and convective hot air drying of sweet gourd. To observe the effect of drying temperature (50 °C, 60 °C, 70 °C) on drying time, sweet gourd slices osmotically treated with 20% salt solution were dried in a mechanical dryer. They observed that when the drying temperature was increased, the drying rate also increased, resulting in a decrease in the necessary drying time.

Regarding the assessment of different drying equations, correlation coefficient (R^2^), SSE (sum of squared error), root means square error (RMSE) and reduced Chi square (χ^2^) were used as the criteria for the accuracy of the fit. The results of parameter estimation for the alternative mathematical models of mushroom drying are presented in Table 2 and Table 3 presents the results of the statistical performance when fitting the experimental data to the thin-layer drying models listed in Table 1. For all six equations investigated, it appears that the R^2^ values are closely approaching a value of 1 for all sample groups, suggesting a good fit of the mathematical models. More specifically, as seen in this table, all the six drying models yielded a correlation coefficient (R^2^) much greater than the acceptable R^2^ value of 0.93 [66] for all different mushroom categories, also showing acceptable values for all the statistical parameters calculated. Among the six drying models, the Midilli, Page, Modified Page, and Weibull models yielded slightly lower values of RMSE and Chi square, with the other equations also presenting a very good fitting performance. The results are in agreement with other researchers who reported the Midilli model to be the most suitable for drying Pleurotus ostreatus [35], Shiitake mushrooms and Jinda chili [34], tomatoes [52], saffron [67], and spearmint [68]. However, taking into consideration that the Lewis equation is only defined by two parameters, when all the other equations include three (Page, Modified Page, Weibull, and Henderson and Pabis) or five (Midilli) parameters to calculate results, the simplest model of Equation (14), representing a simple first-order model, is deemed satisfactory to describe the drying process (water removal) for all the types of mushrooms studied.

#### 3.1.2. Water Activity Reduction

**Figure 3:**
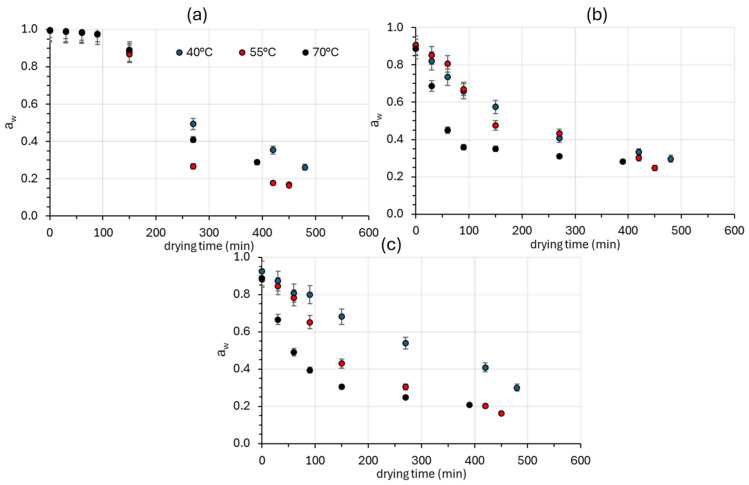
Water activity (*a_w_*) of (**a**) fresh (Control), (**b**) osmotically dehydrated (OD), and (**c**) osmotically dehydrated and previously impregnated with bioactive compounds from *Rosa damascena* distillation wastes (ODR) mushroom samples during hot air drying at 40, 55, and 70 °C. Error bars represent the standard deviation of three measurements.

Water activity (*a_w_*) was found to decrease during drying over time for all sample series, as expected, due to water removal. As shown in Figure 3, there is an inverse relationship between temperature and the equilibrium moisture content at all temperatures studied. This is in agreement with other studies [60]. The control samples exhibit a slower decrease in a_w_ values at all three different temperatures, compared to the osmotically dehydrated counterparts. This difference is more pronounced with increasing temperature, with the largest difference being observed at 70 °C. After 150 min of drying at 70 °C, the a_w_ for the control samples reaches a value of 0.8913 ± 0.0210, while for the osmodehydrated samples, OD and ODR, it is significantly lower, i.e., 0.3512 ± 0.0341 and 0.3054 ± 0.0272, respectively, obtaining values of a shelf-stable product.

### 3.2. Effect of Drying Temperature on Physicochemical Characteristics

#### 3.2.1. Color Parameters

Color is an important sensory attribute for product marketing as it is typically the first characteristic noticed by potential consumers. Additionally, color is often linked to product quality, as it can indicate the level of deterioration in fresh food [13]. In general, color degradation increases with longer drying times and higher temperatures, resulting in greater variation in the color of dried foods [60]. Nevertheless, the color kinetics of foods is a complex phenomenon determined by a variety of factors, including processing conditions, the presence or absence of pigments, and the Maillard reaction, amongst others [69].

**Figure 4:**
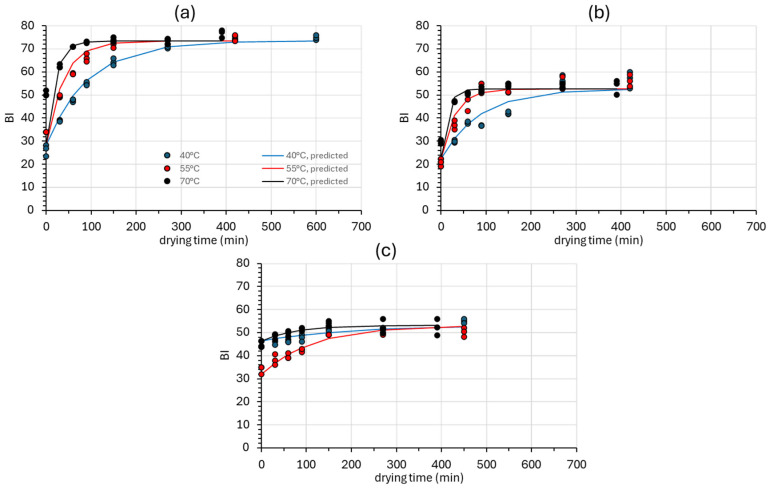
Browing index (*ΒΙ*) increase in (**a**) fresh (Control), (**b**) osmotically dehydrated (OD), and (**c**) osmotically dehydrated and impregnated into *Rosa damascena* by-products (ODR) mushroom samples at 40, 55, and 70 °C. Markers represent experimental data points (triplicates), and solid lines represent the model fitting (Equations (20) and (23)).

The browning of edible mushrooms is a complex process influenced by various intrinsic (i.e., moisture content, respiration rate, and microbial activity) and extrinsic factors, such as process/storage temperature, relative humidity, and mechanical damage. These combined effects lead to cellular damage, enabling substrates to be catalyzed and oxidized into brown pigments by PPO, thus inducing browning. Therefore, PPO might be considered as the primary cause of browning in edible mushrooms [70]. Figure 4 depicts the *BI* change during drying, expressing the organoleptically perceived color darkening, as well as the prediction accomplished through Equations (20) and (23), for all categories of samples investigated. The browning index (*BI*) can comprehensively account for the extent of the brown color on the surface of mushrooms [71]. As shown in Table 3, the overall model (combining Equations (20) and (23)) exhibits satisfactory fitting on the experimental data. The browning index (*BI*) reflects the purity of the brown color and indicates the degree of browning in food materials containing sugar. A lower *BI* value indicates less browning, while a higher value indicates a greater browning of the food material [46]. At the beginning of the air drying process, the mushroom pieces have an average *BI* value of 35.49 ± 11.26 for the control samples, 21.17 ± 6.70 for the OD samples, and 46.81 ± 3.61 for the ODR samples (Figure 4). It is observed that the samples that have been immersed in the rose by-products have higher *BI* values at all three temperatures. This is expected since the rose wastewater had a light brownish color that slightly altered the color of the mushrooms prior to drying. Additionally, there is a significant standard deviation in *BI* values between the sample series (control and OD) due to the significant variability in the raw materials, the unavoidable heterogeneity of the samples at time zero, and the rapid post-harvest deterioration [6]. The ODR samples do not show this difference, as immersion in the wastewater resulted in a uniform color of the samples.

After a few minutes of air drying, both the control and OD/ODR samples reach a browning value equilibrium value. The control samples have a final average value of 73.08 ± 3.46, while the OD samples have a final average value of 50.90 ± 4.42. The *BI* final values are similar at all three different drying temperatures; however, as the drying temperature increases, the equilibrium is achieved at a faster rate. No statistically significant change in browning was observed in ODR at any of the three drying temperatures (*p* > 0.05), with a final *BI* value of 51.62 ± 2.41. The final values of osmodehydrated samples, OD, and ODR are similar. Low *BI* values directly imply a better quality of the dried product [72].

**Figure 5:**
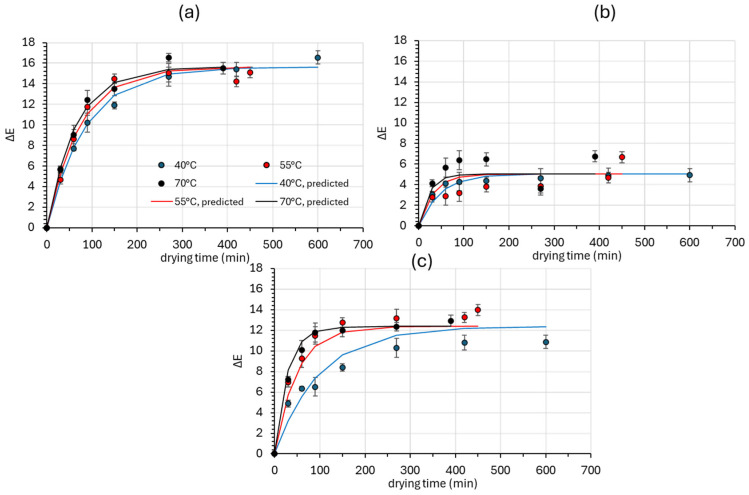
Total color change (Δ*Ε*) of (**a**) fresh (Control), (**b**) osmotically dehydrated (OD), and (**c**) osmotically dehydrated and impregnated into *Rosa damascena* by-products (ODR) mushroom samples at 40, 55, and 70 °C. Markers represent experimental data points, and solid lines represent the model fitting (Equations (21) and (24)). Error bars represent the standard deviation of three measurements.

On the other hand, the total color change (Δ*E*) is shown in Figure 5, where the performance of the prediction model (Equations (21) and (24)) is also illustrated via the solid lines. The parameters of Equations (20), (21), (23) and (24), describing color changes during drying for all categories of mushrooms, are provided in Table 4, along with their 95% confidence intervals. The color change (Δ*E*) is used to characterize the variation in initial color values as a function of processing treatments [46]. The total color change values of the samples increase with increased drying times and air temperatures. Engin [60] also reported higher rates of total color change at 65 °C and the lowest rate at 45 °C, and Guo et al. [73] reported that the color of B. edulis was significantly influenced by the drying temperature. The increase in the overall color difference values may have been caused by non-enzymatic browning, such as the Maillard reaction and sugar caramelization, due to the high temperature applied during drying and the low moisture content [30]. Fresh mushrooms underwent the most pronounced color change compared to the osmotically treated ones at all three drying temperatures. A minor color change was observed in the OD samples, followed by samples treated with *Rosa damascena* distillation wastewater. In general, the OD dried samples at 40 °C showed the highest color retention. Nudar et al. [74] also found that, in terms of quality, osmodehydrated adajamir fruit samples showed a better retention of all quality parameters when compared to the untreated samples.

#### 3.2.2. Texture

**Figure 6:**
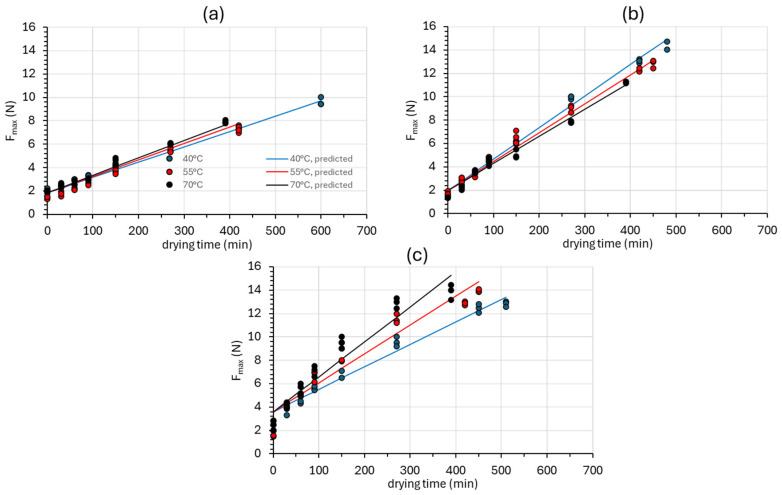
Hardness (*F_max_*) increase in (**a**) fresh (Control), (**b**) osmotically dehydrated (OD), and (**c**) osmotically dehydrated and impregnated into *Rosa damascena* by-products (ODR) mushroom samples at 40, 55, and 70 °C. Markers represent experimental data points (triplicates), and solid lines represent the model fitting (Equations (22) and (25)).

Figure 6 depicts the hardness values (*F_max_*) of the samples during the drying process, also expressing the organoleptically perceived texture change as well as the prediction accomplished through Equations (22) and (25), for all categories of samples investigated. As shown in Table 3, the overall model (combining Equations (22) and (25)) exhibits a satisfactory fitting on the experimental data. The hardness of all samples increased as the drying time increased (Figure 5). The osmodehydrated samples exhibited higher hardness values compared to the control samples. The increased hardness of the OD and ODR samples is probably due to the solid gain that occurred during osmotic dehydration. Regarding the effect of the drying temperature, there is no observed correlation between the drying temperature and the hardness of the samples. At this point, one can also notice the significant variation in the triplicate texture measurements from the range of values depicted in Figure 6, owing to the variability in the raw material and the rough surface of the mushroom samples.

## 4. Conclusions

In this work, the impact of an osmotic dehydration step prior to air drying was investigated for mushroom slices, a subject currently underexplored in the literature. The air drying of OD pretreated and fresh mushroom slices at different process temperatures was kinetically studied and different thin-layer drying models were comparatively assessed, with the simplest first-order Lewis model, including only two parameters, adequately describing moisture ratio decrease. Based on the well-fitted kinetic models of the quality indices’ evolution, the results show that osmotic dehydration prior to hot air drying significantly reduced the browning rate of mushrooms and contributed to a better preservation of their overall color, especially at higher drying temperatures (70 °C). Overall, the results show that the osmotic pretreatment resulted in a significantly reduced drying time at all air drying temperatures, achieving the goal of less energy consumption, along with an improved retention of mushroom texture and color. Additionally, the ODR samples, being enriched with bioactive compounds, have an additional nutritional benefit, assuming they retained their phenolic compounds during air drying [75,76,77]. Nonetheless, to confirm the superior value of the nutritionally “fortified”, osmotreated dried mushrooms, a systematic sensory evaluation is necessary to test the level of correlation between the instrumental determinations with the organoleptic perception. Concluding this study, a dehydrated novel mushroom snack with improved quality attributes and superior nutritional value could be designed and produced through an appropriate manipulation and optimization of both OD and air drying parameters. Nonetheless, one should note that although OD offers multiple advantages, there are currently some important challenges to be addressed and investigated, e.g., long processing time, solute uptake, and reuse of the osmotic solution, before its application is feasible at an industrial scale [78,79]. Furthermore, there is a need for more research on OD conditions to fortify, improve functionality, and incorporate nutraceutical attributes into food matrices to make them attractive for the food industry [80].

## Figures and Tables

**Table 2:** Results of parameter estimation, with ±95% confidence limits, for alternative mathematical models (Table 1, Equations (14)–(19)) of air drying kinetics of fresh and OD/ODR mushroom samples, through non-linear regression analysis.

Parameter	Sample Category		
	Control	OD	ODR
		**Lewis model (Equation (14))**	
***k*_1,*MR*_**	1.2807 ± 0.0286	1.7827 ± 0.0613	1.6728 ± 0.0907
***k*_0,*MR*_**	0.00938 ± 0.00006	0.01042 ± 0.00015	0.01284 ± 0.00027
		**Page model (Equation (15))**	
***k*_1,*MR*_**	1.3578 ± 0.0975	1.6866 ± 0.0632	1.6309 ± 0.0169
***k*_0,*MR*_**	0.0068 ± 0.0002	0.0139 ± 0.0012	0.0144 ± 0.0019
***n***	1.0688 ± 0.0169	0.9359 ± 0.0184	0.9719 ± 0.0304
		**Modified Page model (Equation (16))**	
***k*_1,*MR*_**	1.2703 ± 0.0181	1.8021 ± 0.0001	1.6781 ± 0.0921
***k*_0,*MR*_**	0.00940 ± 0.00004	0.0104 ± 0.0012	0.0128 ± 0.0003
***n***	1.0688 ± 0.0069	0.9358 ± 0.0164	0.9719 ± 0.0204
		**Henderson and Pabis model (Equation (17))**	
***k*_1,*MR*_**	1.2733 ± 0.0217	1.7946 ± 0.0611	1.6734 ± 0.0898
***k*_0,*MR*_**	0.00970 ± 0.00006	0.0102 ± 0.0002	0.0131 ± 0.0004
***n***	1.0256 ± 0.0035	0.9925 ± 0.0093	1.0119 ± 0.0147
		**Weibull model (Equation (18))**	
***k*_1,*MR*_**	−1.2703 ± 0.0181	−1.8021 ± 0.0599	−1.6781 ± 0.0921
***k*_0,*MR*_**	106.21 ± 0.45	96.36 ± 1.37	78.04 ± 1.69
***n***	1.0688 ± 0.0068	0.9359 ± 0.0184	0.9719 ± 0.0304
		**Midilli model (Equation (19))**	
***k*_1,*MR*_**	1.3367 ± 0.0226	1.3367 ± 0.0226	1.5918 ± 0.1034
***k*_0,*MR*_**	0.0076 ± 0.0004	0.0122 ± 0.0019	0.0164 ± 0.0036
***n***	1.0469 ± 0.0118	0.9729 ± 0.0965	0.9585 ± 0.0477
***b***	−1.20 × 10^−5^ ± 0.05 × 10^−5^	6.90 × 10^−5^ ± 0.24 × 10^−5^	6.10 × 10^−5^ ± 0.32 × 10^−5^
***a***	1.0083 ± 0.0044	1.0017 ± 0.0137	1.0342 ± 0.0214

**Table 3:** Statistical results obtained from the selected models of Table 1.

Model	CONTROL				OD				ODR			
	*χ* ^2^	*SSE*	*RMSE*	*R* ^2^	*χ* ^2^	*SSE*	*RMSE*	*R* ^2^	*χ* ^2^	*SSE*	*RMSE*	*R* ^2^
Lewis	0.000232	0.075	0.01517	0.999	0.001029	0.328	0.031985	0.990	0.002072	0.655	0.04538	0.990
Page	0.000099	0.032	0.009922	0.999	0.00091	0.289	0.030018	0.991	0.002059	0.649	0.045161	0.981
Modified Page	0.000098	0.031	0.009920	0.999	0.00091	0.290	0.030043	0.991	0.002059	0.649	0.045161	0.980
Henderson and Pabis	0.000139	0.045	0.011719	0.999	0.000991	0.315	0.031337	0.990	0.002063	0.650	0.045206	0.981
Weibull	0.000099	0.032	0.009922	0.999	0.00091	0.289	0.030018	0.991	0.002059	0.649	0.045161	0.980
Midilli	0.000100	0.032	0.00925	0.999	0.001007	0.318	0.031489	0.991	0.002033	0.636	0.044734	0.981

**Table 4:** Results of parameter estimation, with the ±95% confidence limits, for mathematical models of browning, total color change, and hardness increase during the air drying of fresh and OD/ODR mushroom samples (Equations (23)–(25)) through non-linear regression analysis.

Type of Samples	Parameters of Quality Degradation Indices		
		**Browing Index**	
	***k*_1,*BI*_**	***k*_0,*BI*_**	***R* ^2^**
**Control**	2.78015 ± 0.83306	0.02601 ± 0.00707	0.985
**OD**	3.2572 ± 0.9832	0.03236 ± 0.00986	0.984
**ODR**	1.60568 ± 0.33306	0.00867 ± 0.00096	0.973
		**Δ*E***	
	***k*_1,Δ*E*_**	***k*_0,Δ*E*_**	***R* ^2^**
**Control**	0.54885 ± 0.03533	0.01391 ± 0.00153	0.997
**OD**	1.3306 ± 0.5465	0.03182 ± 0.00676	0.952
**ODR**	2.27573 ± 0.78228	0.02069 ± 0.00185	0.991
		**Hardness (*F_max_*, N)**	
	***k*_1,*F*_**	***k*_0,*F*_**	***R* ^2^**
**Control**	0.2247 ± 0.056	0.014101 ± 0.000616	0.994
**OD**	0.2652 ± 0.0884	0.02751 ± 0.00073	0.996
**ODR**	0.7856 ± 0.0862	0.02472 ± 0.00159	0.987

## Data Availability

The raw data supporting the conclusions of this article will be made available by the authors on request.
